# Sensitive to Infection but Strong in Defense—Female Sex and the Power of Oestradiol in the COVID-19 Pandemic

**DOI:** 10.3389/fgwh.2021.651752

**Published:** 2021-05-11

**Authors:** Louise Newson, Isaac Manyonda, Rebecca Lewis, Robert Preissner, Saskia Preissner, Ute Seeland

**Affiliations:** ^1^Newson Health Ltd, Winton House, Stratford-upon-Avon, United Kingdom; ^2^Department of Obstetrics and Gynaecology, St George's University Hospitals NHS Foundation Trust, London, United Kingdom; ^3^Institute of Physiology and Science-IT, Charité - Universitätsmedizin Berlin, Corporate Member of Freie Universität Berlin, Humboldt-Universität zu Berlin, and Berlin Institute of Health, Berlin, Germany; ^4^Department Oral and Maxillofacial Surgery, Charité–Universitätsmedizin Berlin, Corporate Member of Freie Universität Berlin, Humboldt-Universität zu Berlin, and Berlin Institute of Health, Berlin, Germany

**Keywords:** COVID-19, hormone therapy, mortality, sex, oestrogen, ACE2, long covid

## Abstract

The incidence of SARS-CoV2 infections is around 15% higher in premenopausal women compared to age matched men, yet the fatality rate from COVID-19 is significantly higher in men than women for all age strata. Sex differences have also been observed in recent epidemics including severe acute respiratory syndrome (SARS) and Middle East respiratory syndrome (MERS), with SARS-CoV 2 virus infection sex differences appear more dramatic. The regulation and expression of the angiotensin converting enzyme 2 (ACE2) is the key for this special coronavirus SARS-CoV-2 to enter the cell. 17β-oestradiol increases expression level and activity of angiotensin converting enzyme-2 (ACE2) and the alternative signaling pathway of Ang II via the angiotensin II receptor type II (AT2R) and the Mas receptor is more dominant in female sex than in male sex. Maybe a hint to explain the higher infection risk in women. The same hormonal milieu plays a major role in protecting women where morbidity and mortality are concerned, since the dominant female hormone, oestradiol, has immune-modulatory properties that are likely to be protective against virus infections. It is also known that the X chromosome contains the largest number of immune-related genes, potentially conferring an advantage to women in efficient immune responsiveness. Lifestyle factors are also likely to be contributory. Premenopausal women could possibly face higher exposure to infection (hence higher infection rates) because economic conditions are often less favorable for them with less opportunity for home office work because of jobs requiring mandatory attendance. Due to the additional task of childcare, it is likely that contact times with other people will be longer. Women generally make healthier lifestyle choices, thus reducing the disease burden that confers high risk of mortality in COVID-19 infected men. This narrative review aims to present key concepts and knowledge gaps on the effects of oestrogen associated with SARS-CoV2 infection and COVID-19 disease.

## Introduction

**Figure 1:**
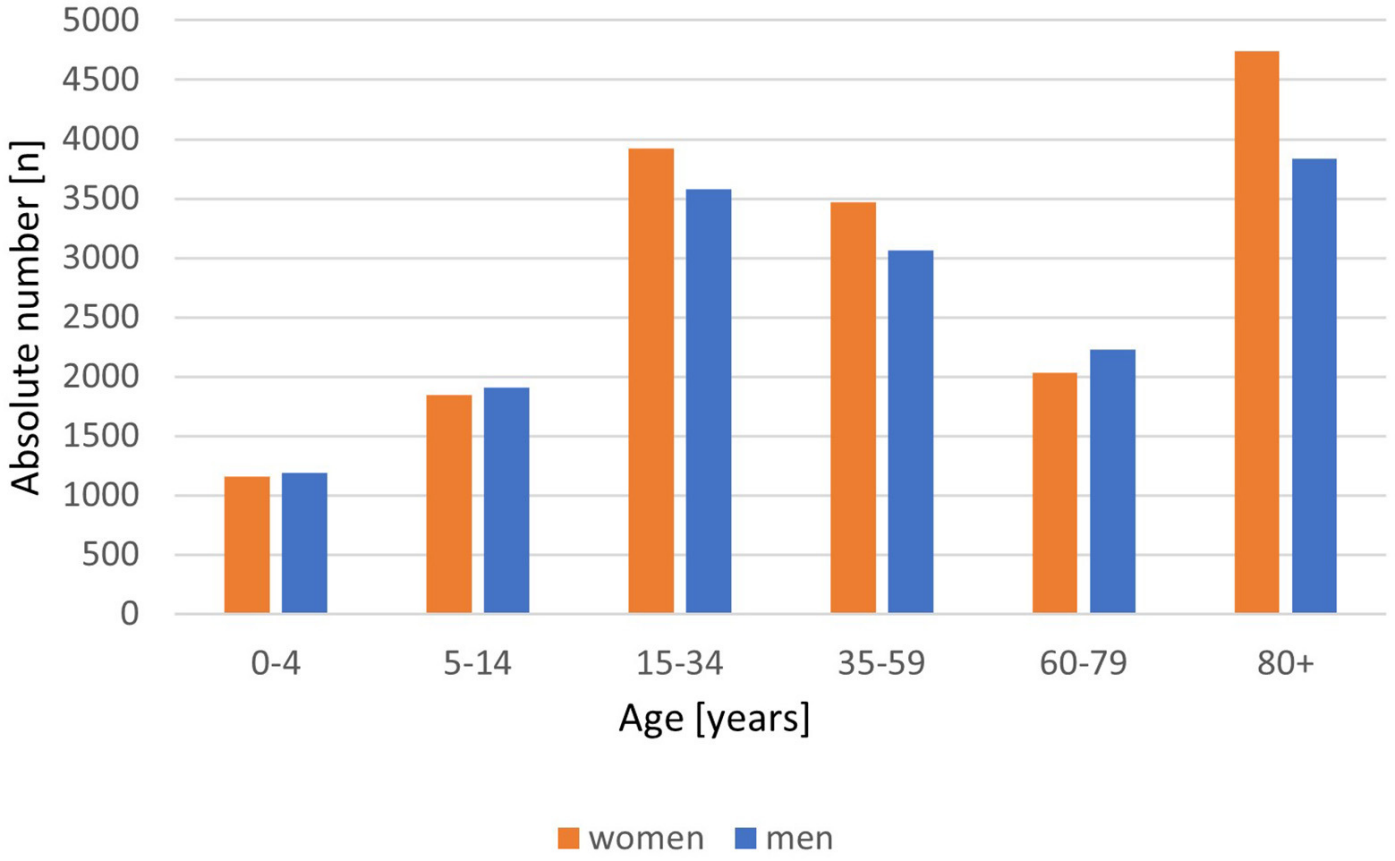
COVID-19 cases by age group and sex/100.000 inhabitants. Absolute number/100.000 inhabitants with COVID-19, disaggregated by women (red) and men (blue). From Robert Koch-Institute/Germany: COVID-19-Dashboard. (retrieved on March 8, 2021).

**Figure 2:**
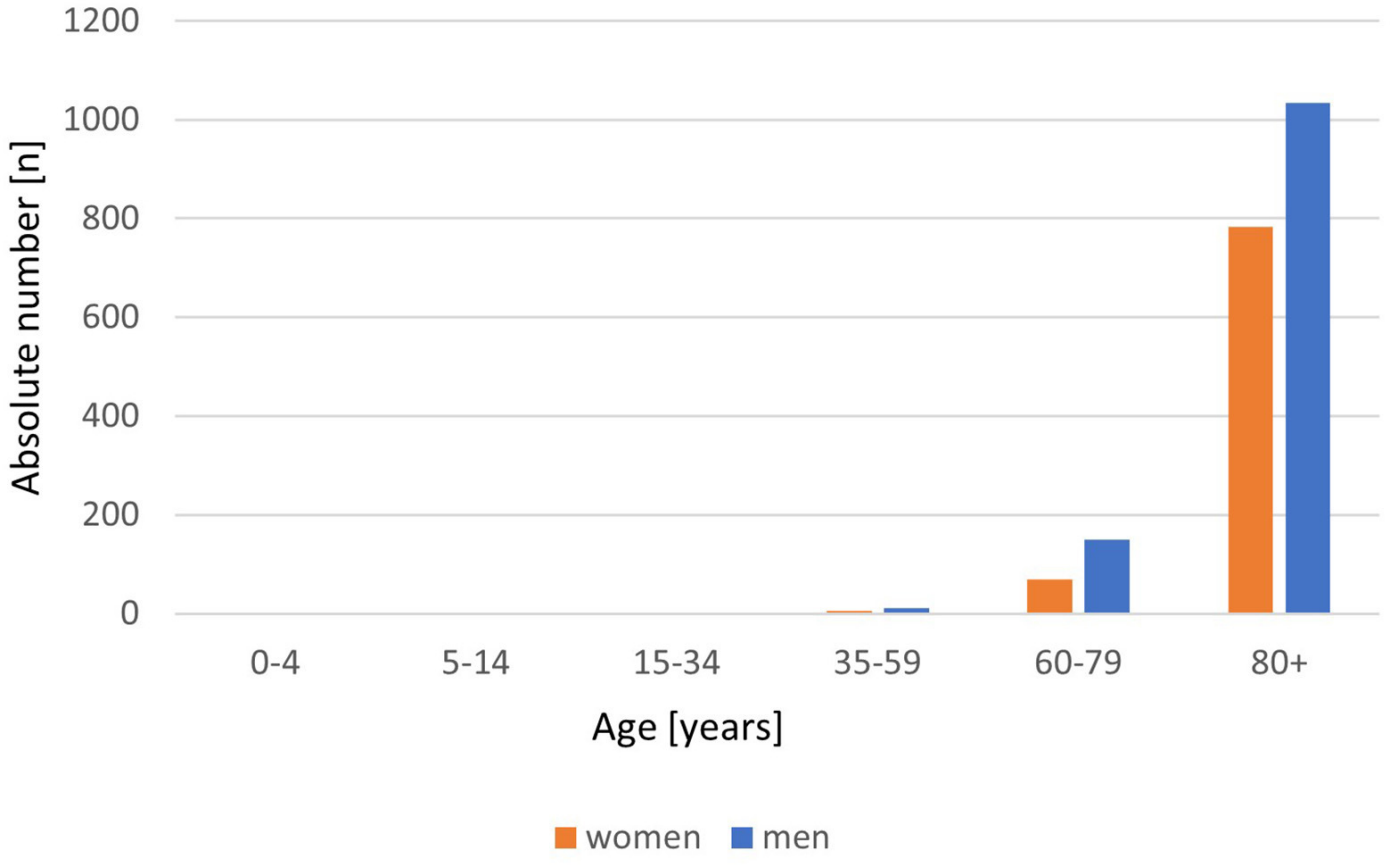
COVID-19 deaths by age group and sex/100.000 inhabitants. Absolute number/100.000 inhabitants of COVID-19 deaths, disaggregated by women (red) and men (blue). From Robert Koch-Institute/Germany: COVID-19-Dashboard. (retrieved on March 8, 2021).

There are marked and intriguing sex differences in infection rates, morbidity, and mortality from COVID-19. Data from our recent study indicate that premenopausal women are disproportionately (15%) more infected with coronavirus than men in the same age brackets, but they do not become as seriously ill, with 50% more men than women dying from this pandemic. (1) The current epidemiology data from Germany (Figures 1, 2) continue to show these sex differences with more SARS-CoV-2 virus infections in premenopausal women and less COVID-19 deaths compared to men in all age strata. This is an interesting observation for sex and gender medicine experts, raising a number of as yet unanswered questions. For example, do the higher infection rates in premenopausal women reflect socio-cultural conditions such as jobs with fewer home office opportunities and more childcare work with increased contacts with and exposure to other families? Could lifestyle factors, such as higher drinking and smoking rates among men, that increase the disease burden (cardiovascular disease, chronic lung disease) play a significant role in the increased death rates in men? Are biological factors in fact more important? There is a profound difference in the hormonal milieu between men and premenopausal women, the dominant female hormone 17ß-oestradiol apparently playing a central role. Studying women with postmenopausal hormone therapy and COVID-19 disease is an additional approach to gain more data to understand oestradiol effects on disease progression. A study from Wuhan has shown that women with low oestradiol levels had more severe infection with COVID-19 (2). Serum 17ß-oestradiol levels are naturally low in men and postmenopausal women. It is noteworthy that there are a variety of mechanisms by which 17ß-oestradiol could impact on outcomes of SARS-CoV-2 infection. It is a potent immune modulator, with both the innate and adaptive immune systems affected, usually but not always in a favorable manner. Within the whole human genome, the X-chromosome contains the largest number of immune-related genes (3), and women possess two of these chromosomes vs. one in men, giving women a theoretical advantage by the phenomenon of X gene escape of the second X chromosome regularly inhibited in function. There are well-documented sex differences in immune responsiveness (4), with women generally mounting better responses when compared to men. Oestrogen is also known to possess/exert non-immune based antiviral activity. It is also conceivable that 17ß-oestradiol also exerts beneficial effects in women via its protection against coagulopathies (5, 6). Hypercoagulability with fibrin formation and polymerization leads to severe COVID-19 disease with thromboembolism and poorer outcomes, especially in men. Last but by no means least, the cardiovascular beneficial effects of oestradiol could also play a central role. The regulation and expression of the angiotensin converting enzyme 2 (ACE2) is the key for this special coronavirus SARS-CoV-2 to enter the cell. Thus, there are strong indications that the dominant female hormone oestrogen is a key player in the protection of women against COVID-19, which are evident from the epidemiological data. Exploring the potential role of oestradiol when used in postmenopausal hormone therapy with more than 50% reduction in mortality in women 50+ with hormone therapy compared to women 50+ without is an interesting starting point to discuss potential mechanisms.

## Review Methodology

In this narrative review, we aim to present key concepts and knowledge gaps identified by the authors on the effects of oestrogen associated with SARS-CoV2 infection and COVID-19 disease. This review includes a selection of the most recent literature, focused on sex and gender differences of SARS-CoV2 incidence and COVID-19 mortality. Based on the existing knowledge, we present a hypothesis on the biological mechanisms that might explain the sex differences known from epidemiological datasets.

### Epidemiology—SARS-COV-2 Infection and COVID-19 Mortality

Epidemiology data on sex differences on severity and mortality in patients with COVID-19 were published by several scientists with almost the same results. For example, Yanez at al. analyzed data for confirmed cases and death from 16 countries and showed drastically increased mortality rates ≥65 years of age with 1.77-fold higher mortality rate in men than in women. (7) A meta-analysis of 58 studies showed men with a 1.57-fold higher odds ratio for mortality and a 1.65-fold higher for severe infection than women (8). Recently Sha et al. confirmed again that mortality of women is lower than in men but noticed no-difference in in-hospital mortality in women < 55 years of age compared with the same age men (9). Beside several limitations of this study like retrospective, exploratory, no measurement of oestrogen level and no information of the history of hormone therapy, they opened the discussion whether an association of oestradiol and mortality in COVID-19 disease exists.

Pro arguments for the lower mortality of women are the oestrogen-mediated low inflammatory response and the gender-related arguments which result in a higher mortality rate in older men than in women like lifestyle, dyslipidaemia, more chronic diseases, and lower lymphocytes. Moreover, our group provided a retrospective analysis of a TriNetX Real-World database contributing the hypothesis of a positive effect of oestradiol to the outcome of COVID-19 disease. The analysis of electronic health records of 68,466 COVID-19 positive patients from 17 countries showed, among other results, a significantly decreased fatality rate of postmenopausal women 50+ with regularly taking hormone therapy with 17ß-oestradiol vs. postmenopausal women without therapy. (1) This effect on fatality rate could not be confirmed for premenopausal women with oral contraceptives vs. non-users. The main result in this premenopausal age group was the 15% higher incidence of SARS-Cov2 infection in women than in men. Similar results for the incidence and the fatality rate are shown by the daily updated statistics published by the Robert Koch Institute (RKI) for Germany, the government's central scientific institution in the field of biomedicine (Figures 1, 2).

### Behavior and Lifestyle—Impact on Gender Differences in COVID-19 Outcomes

For a highly contagious infectious disease such as COVID-19, it is teleologically sound to suppose that behavior and lifestyle could influence outcomes. It has been suggested that male behavior patterns with a tendency to go out into more crowded places such as pubs, a higher use of public transport to access workplaces, and the fact that men tend to wash their hands less frequently than women following high risk exposure (10) would increase their exposure and therefore the incidence of infection. However, evidence shows that women have a 15% higher infection rate than men (1). More studies are needed because possible explanations/mechanisms to explain the different risk profiles between men and women are not fully understood or generalisable. We speculate, admittedly without rigorous research evidence, that during lockdown there might be gender differences in employment patterns that result in more men than women being able to work from home rather than having to go into the office.

It is generally the case that men have jobs that are better paid and have greater flexibility to allow working from home. The burden of parenting responsibilities often falls to women, increasing their exposure if they have to bring their children to the kindergarten and school and then use public transport to attend their places of work. Exposure to infection does not explain the sex differences in morbidity and mortality. However, generally women tend to make healthier lifestyle choices than men: women tend to smoke and drink less than men (11, 12), and consequently often have a lower burden of chronic lung disease or are later in life exposed to cardiovascular disease—that appear to increase mortality risk in COVID-19 patients (13). The challenge is in determining the relative contributions of lifestyle vs. biological factors, and the likelihood is that there is an interplay between the two. This information will only be available if it is possible to use both methodical instruments to collect the facts in parallel in a study, both the measurement of the sex differences due to biological factors and the measurement of the sociocultural influencing gender factors at the same time.

### Physiology—Why Oestrogen Matters

Oestradiol and ACE activity—a possible mechanism for increased COVID-19 infection in women vs. men.

Both the circulating and the tissue renin angiotensin aldosterone system (RAAS) play a crucial role in the regulation of kidney, cardiac and vascular physiology. Activation of angiotensin II (Ang II) by angiotensin converting enzyme (ACE) activity and binding to the angiotensin II receptor type I (AT1R) leads to harmful effects such as tissue remodeling, endothelial dysfunction and fibrosis in target organs. Cardiovascular diseases such as hypertension and heart failure are associated with an activated RAAS.

**Figure 3:**
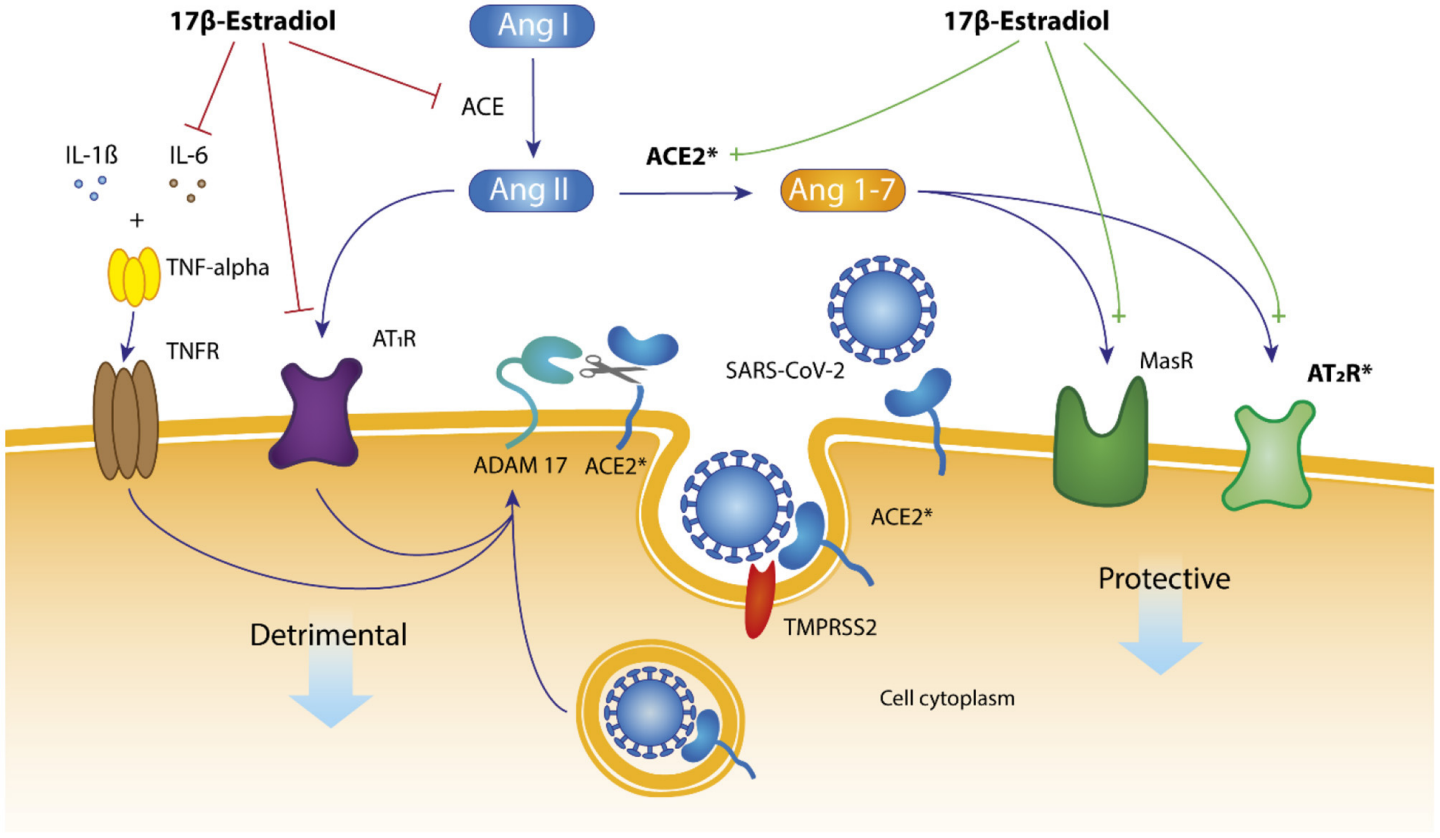
RAAS—Effects of oestradiol on renin-angiotensin-aldosterone system. Hypothesis: mechanistic pathways for the presumably role of 17β-oestradiol for SARS-CoV-2 infection in women. 17ß-oestradiol enhances ACE2 activity. ACE2 cleaves Ang II to Ang1-7 binding to Mas- and AT2 receptor with downstream protective effects for the cell. The membrane-tethered ACE2 protein has an amino- terminal catalytic domain that faces the extracellular space. In case of SARS-CoV-2 infection and endocytosis of ACE2/SARSCoV-2, the metalloproteinase ADAM-17 cleaves membrane bound ACE2 protein followed by downregulation of ACE2 expression at the surface of the cell. The cell thus protects itself against the penetration of further virus particles. Another effect of 17β-oestradiol is the inhibition of IL-6 (interleukin-6) and tumor necrosis factor-α (TNF-alpha) activity followed by less detrimental effects to the cell. ^*^ACE2 and AT2R are located on the X chromosome.

Due to higher 17ß-oestradiol levels another signaling pathway of Ang II via the angiotensin II receptor type II (AT2R) and the Mas receptor is more dominant in women. 17β-oestradiol increases expression level and activity of angiotensin converting enzyme-2 (ACE2) (14). ACE2 cleaves Ang II to Ang 1-7, the substrate for AT2- and Mas receptor. This pathway leads to protective effects on the heart, lung, kidneys, central nervous system and gut (14) (Figure 3). The classical ACE–Ang II–AT1R regulatory axis and the ACE2–Ang 1-7–MasR/AT2R signaling pathway counter-regulate one another. These organ-protective effects of 17β-oestradiol are anti-fibrotic, antioxidant, anti-hypertrophic and vascular dilation effects (14).

ACE2 and the transmembrane protease, serine 2 (TMPRSS2) enzyme, play an essential role in viral entry into host cells and serve as the principal entry receptor for SARS-CoV-2 (15). The membrane-tethered ACE2 protein has an amino-2 terminal catalytic domain (a peptidase) that faces the extracellular space. This protein is expressed in numerous tissues, including the nasal-, respiratory-, intestine-, vascular epithelial cells, kidneys and ovaries. This broad tissue expression of ACE2 enables SARS-CoV-2 to infect nasal endothelial cells and spread to all tissues with ACE2 expression, especially the pharynx and the lung. Cells in the neighborhood of infected cell zones try to protect themselves by changes of intracellular protein expression. However often not for the advantage of the cell in terms of its actual function. Moreover, oestrogens binding to the oestrogen receptor alpha (ERalpha) increase TMPRSS2 expression. In men expression of TMPRSS2 would be associated with the activity of the androgen receptor, which may lead to high expression of TMPRSS2 (16). Another reason for sex differences in addition to the positive effect of 17ß-oestradiol on ACE2 protein expression is the fact that ACE2 and AT2R both being located on the X-chromosome. This results in women to be heterozygous which is clearly different to men, who are hemizygous (17). The second X-chromosome is not inactivated in approximately 15% of genes and another 15% of genes vary in whether they are subject to, or escape from, inactivation (18). This may account for some of the differences that are seen between men and women (sexual dimorphism) and could be a reason for higher expression levels of ACE2- and AT2R proteins in women (i.e., a gene dosage effect).

However, our understanding of the sex-related differences in ACE2 expression in tissues and its levels in plasma is limited, and most of it is based on animal models. Recently, it has been shown that the oestrogen-mediated up-regulation of the Mas-receptor contributes to the prevention of acute lung injury and also improves endothelial barrier stabilization (19). In experimental animal models of acute lung injury from SARS-CoV-2, females have been shown to have some protection compared to males which is likely to be due to the beneficial effects of oestradiol (20); Interestingly, this protection was lost in ovariectomized mice and restored upon oestrogen replacement (21). The 17β-oestradiol molecule has been shown to attenuate lung vascular permeability and oedema, and oestrogen has been shown to reduce the pulmonary vasoconstriction during hypoxia by increasing levels of both prostacyclin and nitric oxide (NO) (22).

Taking all facts presented, the levels of cell-surface–exposed ACE2 generally will be higher in premenopausal women than age matched men and postmenopausal women. Conflicting results were published as well, however, not in cardiovascular health conditions but in animal models or human studies with cardiovascular disease cohorts like higher level of cardiac ACE2 activity in male spontaneously hypertensive rats (SHR) than in female SHR (23).

### Oestrogen and Direct Antiviral Effects

There is accumulating evidence that oestrogen exhibits antiviral activity that is out-with the innate and adaptive immune systems. In an elegant study of transvaginal infection of the simian immunodeficiency virus using ovariectomised macaques, Smith et al. (24) compared the influence of oestrogen vs. progesterone vs. no treatment. None of the oestrogen-treated macaques became infected, while 100% of the untreated and 85% of the progesterone treated became infected following transvaginal inoculation of virus. The researchers were further able to demonstrate that the oestrogen exerted its blocking effect at the level of the vaginal epithelium and/or lumen, since oestrogen-treated macaques became infected following subepithelial inoculation of virus. Johansen et al. have sought to establish if other oestrogen-related drugs could exhibit antiviral activity. Using molecular probes, the team identified a set of selective oestrogen receptor modulators (SERMS)—including clomiphene and toremifene—which acted as potent inhibitors of infection with the Zaire ebolavirus in an *in-vivo* mouse infection model (25). These two SERMS do not appear to inhibit infection through classical pathways associated with the oestrogen receptor, since inhibition occurred even in the absence of detectable oestrogen receptor expression, and both inhibited virus entry after internalization. Instead, the response appeared to be an off-target effect where the compounds interfered with a step late in viral entry and triggering of fusion. In further studies of the mechanism underpinning the antiviral actions of the SERMS, one team of researchers established that the same dosages of SERMs which induced cholesterol accumulation (an incidental biological activity of SERMS) also inhibited Ebola infection. The hypothesis is that SERMs reduced the cellular sphingosine and subsequently caused endolysosomal calcium accumulation, which in turn led to blocking the Ebola virus entry (26). It is a fascinating concept that the simple and innocuous hormone oestrogen could exhibit direct vital antiviral actions that could impact on outcomes in pandemic-prone viruses. This area should be one of intense research activity.

### Oestradiol Effect on Innate and Adaptive Immunity

**Figure 4:**
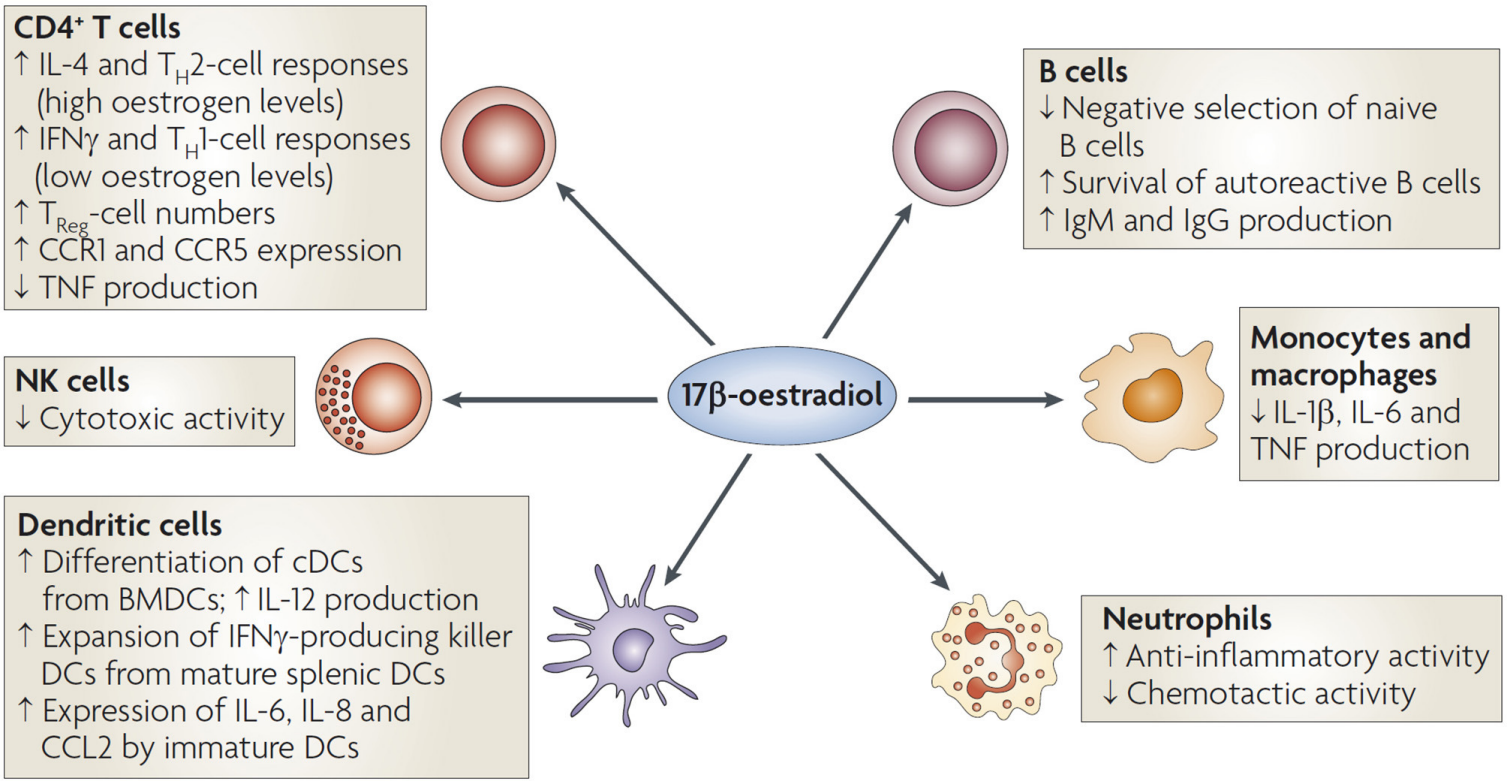
Oestradiol and its effect on immune cells. The activation of oestrogen receptors expressed by T cells, B cells, dendritic cells (DCs), macrophages, neutrophils, and natural killer (NK) cells influences immunocompetence. Low levels of 17β-oestradiol promote TH1-cell differentiation and higher levels promote TH2-cell polarization, with consequent effects on the production of cytokines. Oestrogen decreases tumor-necrosis factor (TNF) production by CD4+ T cells. Oestrogen enhances polyclonal B-cell activation and immunoglobulin production. The inhibition of CD16 expression by oestrogen in monocytes and macrophages leads to the reduced production of the pro-inflammatory cytokines interleukin-1 (IL-1β), IL-6 and TNF. Oestrogen also reduces the cytotoxicity of NK cells (29).

It is well-established that there are differences between sexes in immune responses to infection, with females having better innate and adaptive immune response than males. Sex specific differences are resulting from genetic differences and changing sex steroid hormone levels especially during the menopause transition. Oestrogens regulate both the innate and adaptive response. It can modulate the differentiation, genetic programming and lifespan of all immune cells including neutrophils, macrophages, dendritic cells, and natural killer cells as there are oestrogen receptors (ER) on all these cells (27). The effects of oestrogens on the innate immune responses that are mediated by monocytes and macrophages are largely repressive (28). 17β-oestradiol and its effect on immunocompetence shown Figure 4 (29).

Thinking about sex differences in mortality between women and men with COVID-19, it is important to know, that symptoms will be more severe when the innate and adaptive immune response are strong. That means, for optimal immunological homeostasis to be achieved, the pathogen needs to be removed with high efficiency whilst avoiding collateral tissue damage in the host (30). This immunological balance is known to be different between women and men. Once the immune system is unbalanced in men, it is much harder to return immune response to normal compared to women. We know this phenomenon from other diseases as well, such as glomerulonephritis. Women are known to be able to mount stronger immune responses against viruses and against vaccines. However, they also can exhibit adequate immune-mediated tissue repair capacities (31).

To understand the epidemiological data regarding the discrepancy between the higher incidence of SARS-CoV-2 infections in premenopausal women, and the high fatality rate of men of all ages compared to women, it is important to understand the immune response. Oestradiol has a key role here because it has the ability to reduce the cytokine storm required at the beginning of the infection and to inhibit the cytotoxic NK cells. 17β-oestradiol regulates the production of numerous cytokines and inhibits interleukin (IL)-6 and tumor necrosis factor (TNF)-alpha production. Therefore, 17ß-oestradiol has the potential to attenuate this strong cytokine release which underlies much of the cellular and organ/tissue damage by COVID-19 infection (32).

Past studies have demonstrated that sex has a significant impact on the outcome of infections and has been associated with underlying differences in immune response to infection (33, 34). Previous coronavirus studies (SARS-coronavirus infection) in mice have shown that the female sex hormone oestrogen protected against fatality and lung inflammation. Mice who underwent oophorectomies had more severe disease with more lung inflammation and increased mortality (35). A recent study has linked higher mortality among men to a “cytokine storm,” which in turn closely relates to the severity of symptoms such as pulmonary oedema, fibrosis and other deleterious downstream effects associated with acute lung injury (36). An individual's immune response to viral infections can vary with fluctuations in sex hormone concentrations—oestrogens, progesterone, and testosterone.

Oestrogens at levels of ovulatory phase or pregnancy suppress cytotoxicity of NK cells (37). Notably, macrophages treated *in vitro* with oestradiol showed decreased secretion of the proinflammatory cytokines IL-1β, IL-6, and tumor necrosis factor (TNF)-α (38).

This protective effect, mediated primarily by oestrogen, is attenuated in postmenopausal women. The menopause has a distinct impact on the immune system in women. Postmenopausal women exhibit a reduced number of total lymphocytes, mainly B and CD4+ T lymphocytes (39). Low levels of 17ß-oestradiol can augment inflammatory mediators which could explain the proinflammatory states that most postmenopausal women suffer from (e.g., atherosclerosis) (40). Post-menopausal women are reported to have higher levels of proinflammatory cytokines, such as IL-1β, IL-6, IL-10 and TNF-α (41–44).

However, these levels are reduced with the use of menopause hormone therapy which leads to pre-menopausal levels of oestradiol (45).

### Oestradiol and Long Covid

In women who develop COVID-19, being post-menopausal has been independently associated with more severe infection (46, 47). These effects may be more profound/common in women who are reaching the end of their reproductive life when ovarian function may be more susceptible to viral insult, that is, during the perimenopause and menopause.

The largest group of patients with Long Covid is women in their early 50s. Considering the mounting evidence of interaction between reproductive hormones and COVID-19, the symptoms of Long Covid may be due to the disturbance of physiological ovarian steroid hormone production following COVID-19 and/or an altered chronic inflammatory response due to sex-based immunomodulation during and after the acute infection.

There is evidence that the RAAS is involved in female reproductive processes such as folliculogenesis, steroidogenesis, oocyte maturation and ovulation. Research has confirmed the existence of an Ang-(1–7)–Mas receptor–ACE2 axis and ACE2 markers in all stages of follicle maturation in the human ovary (48).

ACE2 is widely expressed in the ovary and so many patients with Long Covid are experiencing changes in their periods or even their periods stopping is likely to be related. Many of the symptoms such as fatigue, headaches, dizziness, poor concentration, brain fog and memory problems are likely to be a direct consequence of low hormone (oestrogen and testosterone) levels in women.

An online survey of 793 women with Long Covid found that 74% of women reported that their periods have changed since having symptoms of COVID-19. Furthermore, 80% of women reported that their symptoms of Long Covid changed in relation to their menstrual cycle with 78% of women reporting their symptoms being worse prior to or during their periods, when hormone levels are at their lowest (data not published yet).

It is important that there should be greater inclusion of people with Long Covid in clinical trials for potential COVID-19 treatments, including early interventions in the acute phase to prevent long-term complications, and there is a need for more long-term cohort studies of Long Covid (49).

These symptoms are likely to be related to low female hormone levels so consideration should be given as a priority to replacing these low hormone levels with the right dose and type of MHT.

### Treatment Option With Menopause Hormone Therapy in COVID-19 Infection

The menopausal transition provides a unique natural experimental model where the impact of oestrogen on outcomes of COVID-19 infection can be studied, since there is a profound change in the hormonal milieu from the reproductive phase to the menopause. Oestrogen being the dominant hormone that diminishes in the menopause, the experimental design is obvious as this hormone can be administered as HT and its impact on COVID-19 infections studies. With all the evidence presented above pointing to a central role for oestrogen in immune and non-immune response to viral infections, it should not surprise that in women who develop COVID-19, being post-menopausal has been independently associated with more severe infection (46, 47). The largest group of patients with Long Covid (fatigue as the main symptom and physical exhaustion after short period of physical activity) is women in their early 50s, and the symptom profile in these women strongly points to a profound disturbance of physiological ovarian steroid hormone function. Our retrospective analysis of electronic health records of 68,466 COVID-19 positive patients has shown that women taking menopause hormone therapy (MHT) were more than 50% less likely to die from COVID-19 compared to women not taking MHT. This was statistically significant with a Hazard Ratio of 0.29 (95%CI 0.11; 0.76) (1).

A recent UK retrospective cohort study used women with COVID-19 from primary care records found that MHT was associated with a significantly lower likelihood of all-cause mortality in COVID-19 (adjusted OR 0.22, 95%CI 0.05 to 0.94) (50). In addition, there were no reported events for all-cause mortality in women prescribed a combined oral contraceptive pill. The researchers ran multivariable models adjusting for age, ethnicity, index of multiple deprivation, household size, BMI, and comorbidities. They also observed that all-cause mortality risk was higher in COVID-19 amongst women who were older, underweight, from larger households, with hypertension, or on immunosuppressants which is compatible with other studies (50).

We have clear, evidence-based guidelines including from NICE—Menopause: diagnosis and management and from International Menopause Society. Women should be given MHT in the appropriate dose, duration, regimen, and route of administration to improve their symptoms and their future health (51). There is now robust evidence demonstrating that transdermal oestrogen (17-β oestradiol) in association with natural micronized progesterone represents the optimal MHT regimen (52). Transdermal oestrogen is the preferred route of administration because, in contrast with oral oestrogen, oestrogen as a patch, gel or spray is not associated with an increased risk of venous thromboembolism (53). The optimal progestogen is micronized progesterone which is body identical. There is no clot risk with this compared with the older progestogens. In addition, there is no increased risk of breast cancer for at least the first 5 years of taking 17-β oestradiol with micronized progesterone (54, 55).

## Concluding Remarks

The sex and gender differences in favor of women in the morbidity and mortality from COVID-19 infection is well-established, while the underlying mechanisms are open to speculation. The challenge is in determining the relative contributions of lifestyle vs. biological factors, and the likelihood is that there is an interplay between the two. However, the fundamental difference between the two sexes is the hormonal milieu, with oestradiol being the dominant discriminating factor in this regard. This hormone is known to modulate a variety of body functions such as the immune system, viral entry receptors, as well as exhibiting direct antiviral activity, all additively pointing to a crucial role for oestrogen conferring advantages to women in the COVID-19 pandemic. Prospective studies are needed to confirm the positive effect of sex hormone therapy on mortality in postmenopausal women.

## Author Contributions

US wrote the manuscript and designed the figure about mechanistic pathways. LN, IM, RL, RP, and SP helped write the manuscript and added references. All authors read and approved the final version of the manuscript.

## Conflict of Interest

LN and RL are directors of Newson Health Ltd. The remaining authors declare that the research was conducted in the absence of any commercial or financial relationships that could be construed as a potential conflict of interest.
